# The ability of alphavirus replicases to synthesize non-viral type I interferon-inducing RNAs correlates with viral RNA synthesis and has a diverse impact on virus replication and pathogenicity

**DOI:** 10.1128/jvi.02162-25

**Published:** 2026-01-28

**Authors:** Ailar Omler, Anna Rutmane, Suresh Mahalingam, Andres Merits

**Affiliations:** 1 University of Tartu, Institute of Bioengineering 37546, Tartu, Estonia; 2 Emerging Viruses, Inflammation and Therapeutics Group, Institute for Biomedicine and Glycomics, Griffith University 265012, , Gold Coast, Queensland, Australia; 3 Global Virus Network (GVN) Centre of Excellence in Arboviruses, Griffith University 5723, Gold Coast, Queensland, Australia; 4 School of Pharmacy and Medical Sciences, Griffith University 5723, Gold Coast, Queensland, Australia; St Jude Children's Research Hospital, Memphis, Tennessee, USA

**Keywords:** alphavirus, interferons, double-stranded RNA, viral RNA replicase, type I interferon, self-amplifying RNA vaccines, innate immune sensing, chikungunya virus, Semliki Forest virus

## Abstract

Alphaviruses have positive-strand RNA genomes that mimic cellular mRNAs, and their translation results in the synthesis of nonstructural (ns) polyprotein, the precursor of viral replicase. The ns polyprotein is processed by its protease activity to form an early replicase complex, responsible for the synthesis of negative-strand RNA that forms a double-stranded RNA (dsRNA) replication intermediate with the RNA genome. The following processing results in the formation of a late replicase complex responsible for the synthesis of positive-strand RNAs. Replication complexes are anchored to membranes, and dsRNA is shielded from cellular pattern recognition receptors. Nevertheless, alphavirus infection triggers a type I interferon response; this is partly due to the ability of replicases to utilize cellular RNAs as templates for synthesis of specific dsRNAs (rPAMPs). Here, we demonstrate that replicases of 11 alphaviruses, representing most of the antigenic complexes of alphaviruses, are all capable of rPAMP synthesis in human cells and that some replicases also do the same in mosquito cells. The levels of rPAMPs generally correlate with the efficiency of viral RNA synthesis and are increased by mutations slowing down the processing of the ns polyprotein. For different strains of Semliki Forest virus, the elevated synthesis of rPAMPs correlates with a previously reported virulent phenotype, while for mutants of chikungunya virus, the situation is reversed. Thus, synthesis of rPAMPs is a universal property of alphavirus replicases; these molecules are used to regulate virus infection, and their functional impact depends on their amount as well as the virus species.

**IMPORTANCE:**

Alphaviruses are important mosquito-borne emerging pathogens. Their ability to interact with cellular defenses, including type I IFN, is crucial for infection. Here, we found that alphavirus replicases have a universal ability to synthesize type I IFN-inducing RNAs using non-viral templates, and that their synthesis varies greatly among viruses and their strains. Production of these RNAs was increased by mutations slowing down the maturation of the viral replicase. The abundance of non-viral type I IFN-inducing RNAs correlated with neurovirulence of Semliki Forest virus, indicating their role in virus pathogenicity; however, for chikungunya virus, their excess correlated with virus attenuation. These data are important to promote the understanding of mechanisms of alphavirus pathogenesis and virus interactions with the host immune system. As alphaviruses represent promising platforms for development of advanced mRNA vaccines, the data can also be used for rational optimization of alphavirus-based vaccine candidates.

## INTRODUCTION

Alphaviruses (family *Togaviridae*) are enveloped positive-strand RNA viruses (1). The majority of more than 30 known alphavirus species are transmitted between vertebrate hosts by arthropod vectors, most commonly by mosquitos, and often represent important (re)emerging human pathogens. Arthritogenic alphaviruses cause debilitating arthritis and can be found around the globe; in the case of chikungunya virus (*Alphavirus chikungunya,* CHIKV) infection, long-lasting chronic symptoms, persisting for months or years, are common (2). Encephalitic alphaviruses, such as eastern, western, and Venezuelan equine encephalitis virus (*Alphavirus eastern,* EEEV; *Alphavirus western,* WEEV; and *Alphavirus venezuelan,* VEEV), are endemic to the New World and may cause disease with lethal outcome (3). There are no approved antivirals or treatments for alphavirus infection (4, 5).

The alphavirus virion contains a single 10–12 kb RNA genome, with the 5′ region encoding nonstructural (ns) proteins and the 3′ region encoding structural proteins (6). The genomic RNA is capped at the 5′ end and polyadenylated at the 3′ end to mimic host mRNA and contains two open reading frames (ORFs). ORF1 is translated from the genomic RNA and encodes nonstructural (ns) polyproteins P123 and P1234, precursors of four ns proteins (nsP1-4) that are virus-encoded components of the replication complex (RC) (6). P123 and P1234 are expressed immediately after the release of the virus genome into the cytoplasm of infected cells and are processed into functionally important processing intermediates and mature nsPs by the protease activity of nsP2. The processing cascade starts from the release of nsP4, the viral RNA-dependent RNA polymerase (RdRp), resulting in the formation of the early replicase (P123 + nsP4), the negative-strand RNA synthesis complex (7). Negative-strand RNA and genomic RNA form double-stranded (ds) RNA replication intermediates that localize in virus-induced membrane invaginations called spherules (8), where they are shielded from cellular pathogen-associated molecular pattern (PAMP) recognition receptors (PRR). Subsequent processing of P123 first releases nsP1; the event is rapidly followed by the cleavage of the remaining P23 and formation of the late replicase, consisting of individual nsPs, and synthesis of new genomes as well as subgenomic (SG) RNAs used for translation of structural proteins encoded by ORF2 (9). The core of the late replicase complex consists of a ring structure formed by 12 molecules of nsP1, one molecule of nsP4 that is located in the pore of the ring, and one molecule of nsP2 interacting with nsP4 and located at the cytosolic side of the complex (10). These enzymes, involved in viral RNA synthesis and capping, are located at the neck region of spherules and are also associated with a cytosolic ring structure formed by nsP3 and host proteins (10, 11). Interestingly, in alphavirus-infected cells, there are also numerous replicase core structures that lack cytosolic ring structure and are not associated with spherules (10). Thus, these complexes cannot be involved in viral RNA synthesis, and their function(s) remain unknown.

Despite sequestration of viral dsRNA inside spherules (12), cells can detect alphavirus infection. In vertebrate cells, the innate sensing of alphavirus PAMPs triggers type I interferon (IFN) production and signaling to activate expression of interferon-stimulated genes (ISGs) to achieve a substantial antiviral state (13). As the alphavirus RNA genome itself mimics host mRNA, the major PAMPs generated during alphavirus infection are other products of the viral RNA replicase. RIG-I and MDA5 are PRRs that detect cap-deficient, single-stranded RNAs and dsRNA, both of which are intermediates or by-products of alphavirus replication (14). Additionally, membrane-bound Toll-like receptors, TLR3, TLR7, and TLR8, can detect such RNAs in endosomes. The activated PRRs initiate a signaling cascade to activate transcription factors IRF3 and IRF7, which trigger the production of type I IFN (15).

As the activation of the innate immune response hampers infection, viruses have developed multiple counter-mechanisms to downregulate type I IFN production and signaling. In the case of arthritogenic alphaviruses, a fraction of nsP2 localizes into the cell nucleus, where it induces degradation of Rpb1, the catalytic subunit of RNA polymerase II (16). The capsid protein of encephalitic alphaviruses contains both nuclear localization and nuclear export signals, resulting in shuffling between the nuclear and cytoplasmic compartments and in the impairment of nuclear transport of host factors (17, 18). In addition to causing general host cell shutoff, more specific mechanisms of suppression of innate immune responses have also been described (19–21). Despite all these mechanisms to inhibit the host response, alphavirus infection often still results in high levels of type I IFN production, suggesting the possibility that selective activation of the host innate immune response may represent a part of the alphavirus attack strategy. In line with this, it was very recently demonstrated that the type I IFN signaling limits infection of dendritic cells by alphaviruses, resulting in diminished direct antigen presentation; this reduces antiviral CD8^+^ T-cell responses and results in incomplete clearance of Ross River virus (*Alphavirus rossriver*, RRV) infection (22). Therefore, it is logical to assume that alphaviruses have evolved mechanism(s) allowing selective activation of type I IFNs. Semliki Forest virus (*Alphavirus semliki*, SFV), Sindbis virus (*Alphavirus sindbis*, SINV), Barmah Forest virus (*Alphavirus barmah,* BFV), and RRV have been shown to utilize non-viral RNAs as templates for synthesis of specific replicase-generated PAMP RNAs (rPAMP); these molecules are produced not only in virus-infected cells but also in the absence of an amplification-competent viral RNA template, for example, in cells transfected by expression plasmids for ns polyproteins (23–25). Experiments with SFV have revealed that rPAMPs are non-polyadenylated 5′-ppp dsRNAs with duplex length larger than 200 bp. Thus, existing data suggest that the synthesis of rPAMPs may be a general property of RNA replicases of arthritogenic alphaviruses. Furthermore, the previously described pathogenic phenotype of SINV (26) correlates with the ability of its corresponding replicase to synthesize high levels of rPAMPs (24), suggesting their functional importance.

Here, we extended the analysis of the ability of alphavirus replicases to produce rPAMPs to encephalitic and insect-specific alphaviruses. It was found that in human cells, replicases of all these alphaviruses synthesize these type I IFN-inducing RNAs. The efficiency of their synthesis varied among replicases of different alphaviruses and generally correlated with the efficiency of amplification of matching template RNA by the corresponding replicase. Mutations altering the ns polyprotein processing of SFV, CHIKV, and BFV had major impacts on rPAMPs production; in general, synthesis of rPAMPs was facilitated by increased stability of P123. The ability to synthesize high levels of rPAMPs was associated with previously described *in vivo* phenotypes of viral strains and mutants. However, while for SFV, increased virulence was associated with elevated rPAMP synthesis, the reverse was true for CHIKV, indicating that different alphaviruses have different abilities to exploit these molecules. Surprisingly, it was found that replicases of SFV, CHIKV, WEEV, and Mayaro virus (*Alphavirus mayaro,* MAYV) also synthesize similar type I IFN-inducing RNAs in mosquito cells, indicating that these RNAs may also have functions other than induction of type I IFN expression. Overall, this study contributes to the understanding of events that lead to the detection of alphavirus infection by host innate immunity. Our findings have potential use for the design of alphavirus-based therapeutics, including advanced vaccine candidates.

## RESULTS

**Fig 1:**
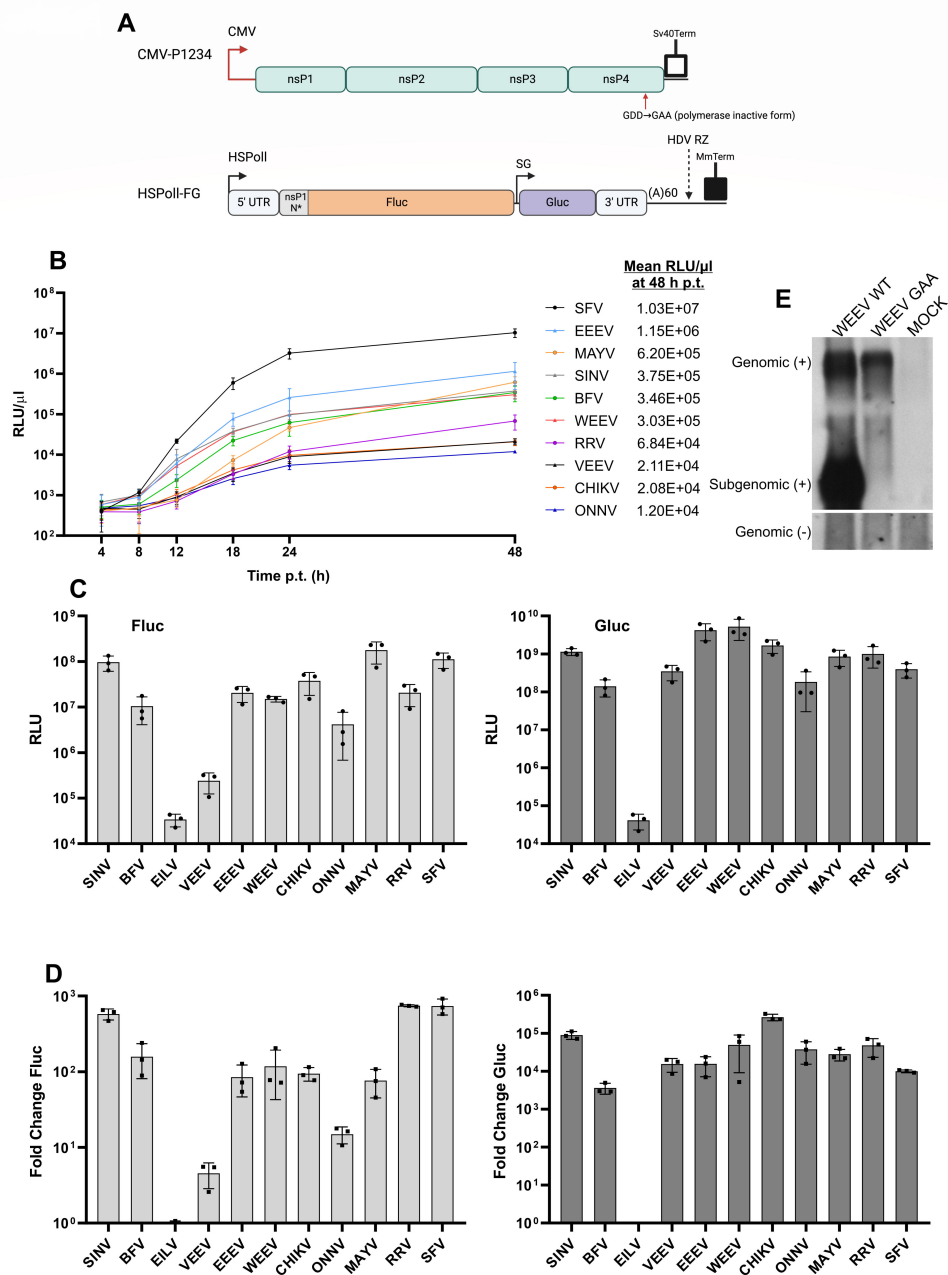
Activities of replicases of different alphaviruses in COP5 and HEK293T cells. (**A**) Schematic presentation of expression constructs of alphavirus replicase and mini-genome for mammalian cells. CMV, immediate early promoter of human cytomegalovirus; Sv40Term, terminator for RNA polymerase II; HSPolI, truncated promoter for human RNA polymerase I; 5′ UTR, 5′ untranslated region; N77, region encoding the N-terminal 77–110 amino acid residues of P1234; SG, subgenomic promoter; 3′ UTR, truncated 3′ untranslated region (last 110 residues); HDV RZ, antisense strand ribozyme of hepatitis delta virus; MmTerm, a terminator for RNA polymerase I in mice. Black arrows designate start sites of genomic and SG promoters. The position of the GDD to the GAA mutation in nsP4 used to inactivate the viral RNA polymerase is indicated. The image was created using BioRender. (**B**) COP5 cells grown on 24-well plates were co-transfected with 0.5 µg of CMV-P1234 and 0.5 µg of corresponding HSPolI-FG plasmids. Samples of growth media were collected at 4, 8, 12, 18, 24, and 48 hours post transfection (hpt). Activities of secreted Gluc are presented as relative light units (RLU) per 1 µL of growth media (RLU/µL). Mean values from three independent experiments are shown. (**C, D**) HEK293T cells grown on 24-well plates were co-transfected with 0.5 µg of CMV-P1234 and 0.5 µg of corresponding HSPolI-FG plasmids; for control cells, CMV-P1234^GAA^ was used instead of plasmid-expressing active replicase. Cells were lysed at 18 hpt, and the activities of Fluc and Gluc were measured. (**C**) Absolute activities of Fluc and Gluc presented in RLU per 20,000 transfected cells (**D**). Activities of Fluc and Gluc in cells expressing active replicases were normalized to those in control cells (taken as 1). For panels C and D, individual data points, mean values, and standard deviation (SD) from three independent experiments are shown. (**E**) HEK293T cells grown in six-well plates were either mock transfected or co-transfected using 2 µg CMV-P1234-WEEV or CMV-P1234-WEEV^GAA^ and 2 µg HSPolI-FG-WEEV. At 18 hpt, total RNA was extracted and analyzed by northern blotting using a probe corresponding to the Fluc reporter gene to detect negative strands (lower panel, “Genomic [-]”) or a probe complementary to the Gluc reporter gene to detect positive strands (upper panel, “Genomic [+], Subgenomic [+]”). Note that an RNA of the same size as “Genomic (+)” is also synthesized by cellular RNA polymerase I and is therefore also detectable in cells transfected using CMV-P1234-WEEV^GAA^, which encodes an inactive replicase unable to utilize the provided RNA template (WEEV GAA). The experiment was repeated two times with similar results; data from one experiment are shown.

Alphaviruses are divided into eight antigenic complexes (27). While they share similar virion architecture, genome expression, and replication strategies, their interactions with hosts are surprisingly different. Alphaviruses utilize different host factors to assist their genome replication (28), use different viral proteins to cause host cell shutoff (18), and, even within the same antigenic complex, use very different entry receptors (29). Previously, synthesis of rPAMPs has been reported for replicases of arthritogenic alphaviruses belonging to the Semliki Forest complex (SFV, RRV), Barmah Forest complex (BFV), and western equine encephalitis complex (SINV) (23–25). Although SINV belongs to the western equine encephalitis complex, its nsPs are evolutionarily derived from viruses distantly related to WEEV and other encephalitic alphaviruses; therefore, we also included the WEEV replicase. Due to the diversity of alphavirus-host interactions, it was unclear if this ability is also shared by alphaviruses belonging to other antigenic complexes. Therefore, we extended the analysis of rPAMP production to replicases of alphaviruses belonging to eastern equine encephalitis (EEEV) and Venezuelan equine encephalitis (VEEV) antigenic complexes, as well as to the insect-specific Eilat virus (*Alphavirus eilat,* EILV); Middleburg complex and Ndumu complex, each containing a single poorly studied member, were excluded from the analysis. To analyze the extent to which the ability to synthesize rPAMPs varies among viruses within the same complex, we also included the replicases of CHIKV, MAYV, and o’nyong-nyong virus (*Alphavirus onyong*; ONNV)—major human pathogens from the Semliki Forest complex. Advantage was taken of previously developed alphavirus replicase expression plasmids and *trans*-replicase systems (Fig. 1A) that permitted the analysis without a need to use highly pathogenic viruses and sophisticated fractionation of type I IFN-inducing RNAs generated during virus infection.

In the absence of template RNAs, plasmids expressing P1234 can be used to produce rPAMPs. However, if the suitable amplification-competent RNA (a mini-genome) is provided, the *trans-*replicases allow assessment of the activities of viral RNA replicase expressed in the form of the P1234 precursor (30) to make the assay simple and sensitive, the amplification-competent RNA is designed to express easily quantifiable firefly luciferase (Fluc; marker of genomic RNA replication efficiency) and *Gaussia* luciferase (Gluc, marker of SG RNA transcription efficiency) reporters. Thus, these tools allowed us to establish the correlation between the ability of replicase to synthesize rPAMPs and its ability to synthesize viral RNAs.

### In human cells, the production of rPAMPs is a universal property of alphavirus RNA replicases

Both the synthesis of rPAMPs and their ability to induce IFN-β expression have been previously analyzed using murine fibroblast (COP5) cell lines transfected with alphavirus P1234 expression plasmids (23, 24). However, several alphaviruses, including CHIKV and ONNV, replicate poorly in mice or in type I IFN-competent murine cells, while other viruses, for example, SFV, replicate to very high titers. Analysis of the expression kinetics of Gluc marker in COP5 cells confirmed that these differences are also reflected in activities of the corresponding *trans*-replicases; at 48 h post transfection (hpt), the difference between Gluc activities produced by *trans-*replicases of ONNV and SFV was ~850-fold (Fig. 1B). In contrast, differences in the activities of Fluc and Gluc markers produced in human HEK293T cells transfected by the *trans*-replicase system (absolute activities) or these normalized to activities of inactive controls (normalized activities) did not exceed 50-fold (Fig. 1C and D); the only exception was the activity of *trans*-replicase of EILV, which was consistently very low. This finding is consistent with previous reports (30, 31) and indicates that HEK293T cells are more suitable than murine cells for comparison of activities associated with RNA replicases of different alphaviruses. It was also observed that the activities of WEEV *trans*-replicase, which has not been previously analyzed, were high and similar to those of the replicase of EEEV (Fig. 1C and D). Consistent with this, high levels of WEEV positive-strand RNAs were detected using northern blot (Fig. 1E).

**Fig 2:**
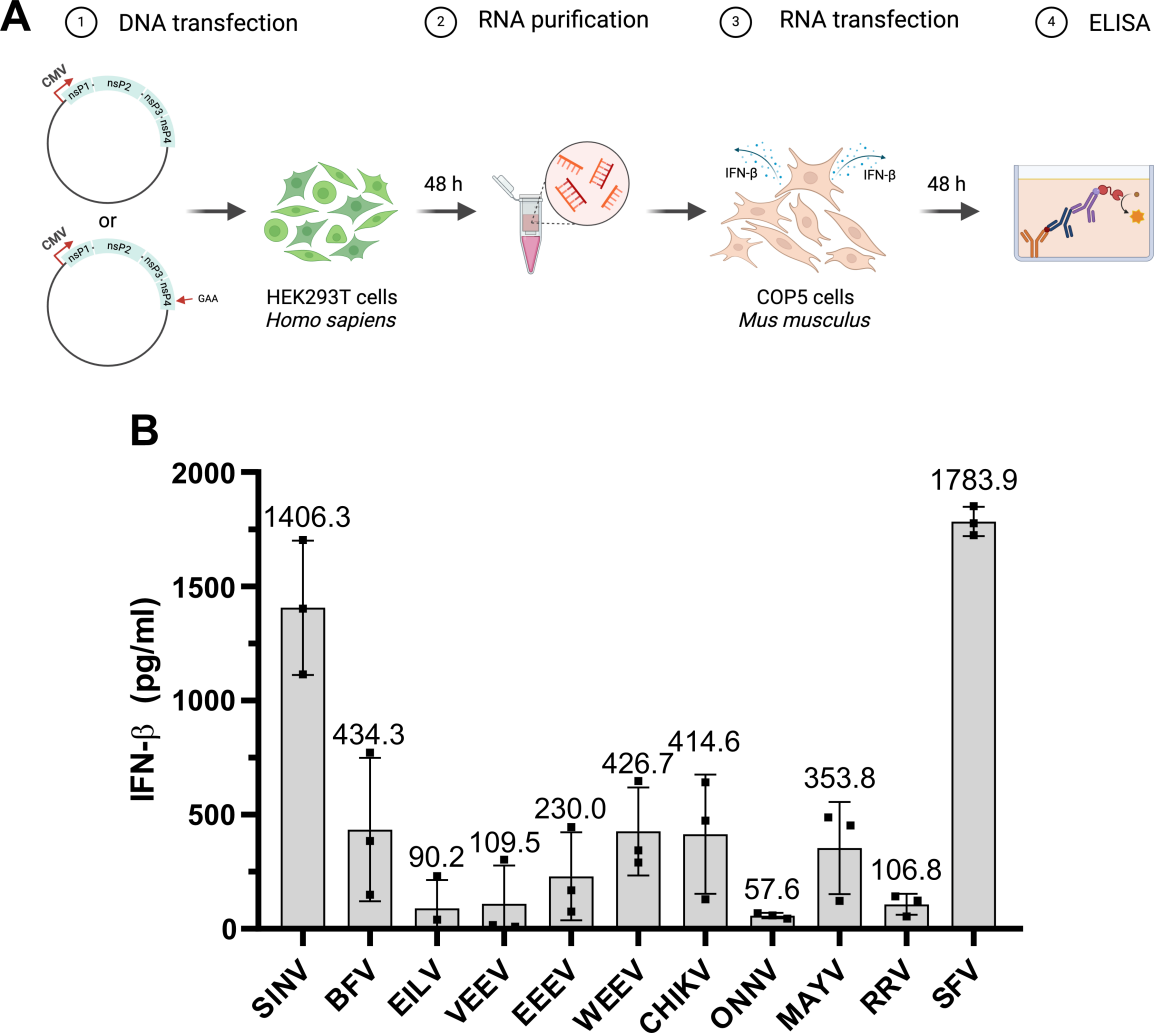
Alphavirus replicases produce type I IFN-inducing rPAMPs in HEK293T cells. (**A**) Schema of experiment used for the detection of rPAMPs produced in HEK293T cells. Cells grown on 24-well plates were transfected with 2 µg of CMV-P1234 or CMV-P1234^GAA^ of each analyzed virus. At 48 hpt, cells were collected, and total RNA was isolated. 5 µg of total RNA was used to transfect COP5 cells, and amounts of secreted IFN-β-cell culture medium were measured at 48 hpt. The image was created using BioRender. (**B**) Induction of IFN-β by RNAs isolated from HEK293T cells transfected with expression plasmids of replicases of the indicated alphaviruses. For each data point, amounts of IFN-β induced by RNAs isolated from CMV-P1234^GAA^-transfected control cells were subtracted from amounts of IFN-β induced by RNAs isolated from cells transfected using corresponding CMV-P1234. Columns represent mean values (shown in numbers) with SD from three independent experiments; individual data points are shown as dots.

In HEK293T cells, the type I IFN induction pathway is only partially functional (32), precluding the use of these cells for direct measurement of type I IFN induction. Furthermore, the abilities of replicase proteins of different alphaviruses to suppress type I IFN induction and signaling are drastically different as nsP2, the major antagonist of type I IFN responses used by arthritogenic alphaviruses, is an integral part of RNA replicase, while the capsid protein, the major antagonist of type I IFN responses by encephalitic alphaviruses, is not. Therefore, we developed a novel two-step assay where the synthesis of rPAMPs was performed in HEK293T cells transfected with P1234 expression constructs while the amounts of rPAMPs were estimated by measuring the ability of purified total RNA from transfected HEK293T cells to induce IFN-β production upon transfection of COP5 cells (Fig. 2A). The most prominent IFN-β induction was observed for RNAs purified from HEK293T cells transfected with plasmids expressing P1234 of SFV or SINV (Fig. 2B); these high type I IFN-inducing abilities are consistent with previous data obtained using COP5 cells (23, 24). Furthermore, these data also correlate with previous findings that *trans*-replicases of SINV and SFV synthesize the highest levels of viral RNAs in HEK293T cells (30). More modest IFN-β induction was observed for RNAs from HEK293T cells expressing replicases of BFV, WEEV, CHIKV, and MAYV. Low levels of IFN-β were induced by RNAs generated by replicases of EILV, VEEV, EEEV, ONNV, and RRV (Fig. 2B). Again, these data mostly correlate with the abilities of corresponding *trans-*replicases to synthesize viral RNAs (Fig. 1C and [30]). The exceptions from this rule were replicases of EEEV and RRV that also produced rather modest levels of IFN-β-inducing rPAMPs (Fig. 2B) despite the fact that in the *trans-*replicase assays, both of these replicases are highly efficient in viral RNA synthesis (30, 31). Taken together, this analysis confirmed that replicases of encephalitic alphaviruses and, surprisingly, even the replicase of insect-specific EILV are capable of synthesizing rPAMPs, indicating that rPAMP synthesis in human cells is a universal feature of alphavirus RNA replicases.

### Replicases of virulent strains of SFV synthesize higher levels of viral RNAs and rPAMP than replicases of avirulent SFV strains

The amounts of rPAMPs synthesized by replicases of different viruses belonging to the Semliki Forest complex differ as much as 30-fold (Fig. 2B). As these viruses have multiple strains with different phenotypes, we also analyzed whether the variation in rPAMP synthesis also extends to different strains of the same virus. SFV strains are classified as avirulent or virulent by the disease severity in adult mice (33). We have previously shown that a virus rescued from an infectious cDNA (icDNA) clone SFV6 (based on the virulent L10 strain) causes lethal encephalitis in mice, while the virus rescued from the icDNA of A774 (based on the avirulent A7(74) strain) does not. The determinants of virulence have been mapped to the nsP3 region as well as to the residues in the P4 position (residue 534 in nsP1) of the cleavage site between nsP1 and nsP2 (1/2 site) and in the S4 position (residue 515 in nsP2) of the corresponding subsite of the nsP2 protease (34).

**Fig 3:**
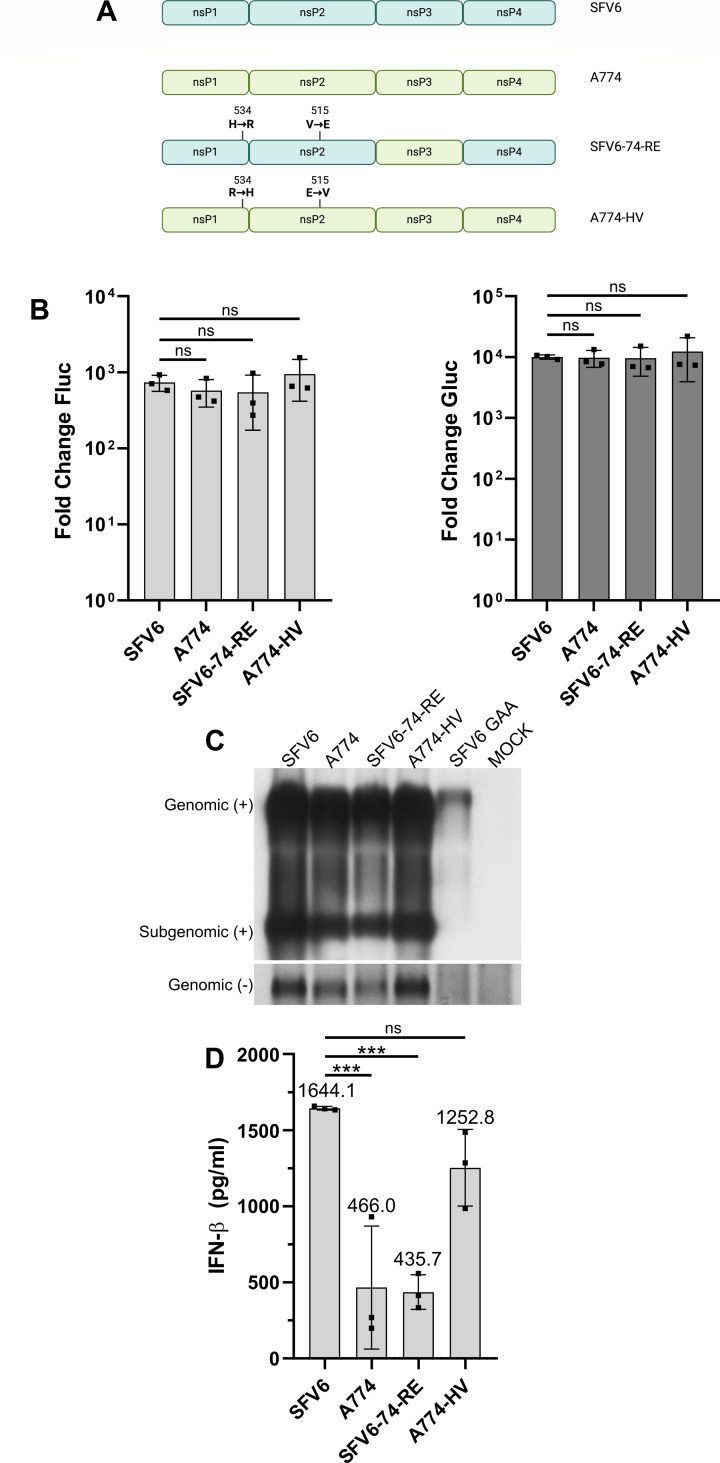
Replicases of virulent SFV strains synthesize higher levels of viral RNAs and rPAMPs. (**A**) Schematic representation of the used SFV ns polyproteins. Point mutations are indicated above the drawings, and the swap of nsP3 is indicated by color code. The image was created using BioRender. (**B**) HEK293T cells grown on 24-well plates were co-transfected with 0.5 µg HSPolI-FG-SFV and 0.5 µg of the indicated CMV-P1234 plasmids; in control cells, the CMV-P1234^GAA^-SFV was used instead of plasmid-expressing active replicase. Data were collected, analyzed, and presented as described for Fig. 1D; ns, not significant (Student’s unpaired *t*-test). (**C**) HEK293T cells grown on six-well plates were either mock transfected or co-transfected using 2 µg CMV-P1234 plasmids or CMV-P1234-SFV^GAA^ and 2 µg HSPolI-FG-SFV. Cells were collected, analyzed, and data are presented as described for Fig. 1E. (**D**) HEK293T cells grown on 24-well plates were transfected with 2 µg of CMV-P1234 of the indicated SFV strains; control cells were transfected with CMV-P1234^GAA^-SFV. The experiment was performed as shown in Fig. 2A, and data were analyzed and presented as described for Fig. 2B. Statistical analysis was performed using one-way ANOVA; ***P < 0.001; ns, not significant.

The replicase of SFV, which originates from the SFV6 strain, had high activities (Fig. 1C and D) and synthesized very high levels of rPAMPs (Fig. 2B). To find out whether these are common properties of replicases of different strains of SFV, we compared the activities of *trans-*replicases, efficiencies of viral RNA and rPAMP synthesis of replicases from virulent SFV6, the avirulent SFV6-74-RE mutant, avirulent A774 strain, and its virulent A774-HV mutant (Fig. 3A). All four *trans*-replicases were highly active, and small differences in activities were not statistically significant (Fig. 3B). The northern blot analysis revealed that replicases of SFV6 and A774-HV synthesized somewhat higher levels of negative-strand RNAs as well as elevated amounts of positive-strand RNAs (Fig. 3C). These SFV strains were previously found to have slower processing of P123 due to delayed cleavage at the 1/2 site (34). Most likely, this results in an increased stability of the early (P123+nsP4) replicase and leads to the increased synthesis of negative-strand RNAs and consecutively also positive-strand RNAs. It was found that the synthesis of rPAMPs had the same pattern: RNAs from HEK293T cells expressing P1234 of SFV6 or A774-HV induced much higher levels of IFN-β than did RNAs from cells expressing P1234 of A774 or SFV6-74-RE (Fig. 3D). These data further highlight the link between the efficiency of viral RNA synthesis and the ability of replicases to generate rPAMPs, such that relatively modest differences in replicase function translate into pronounced differences in the production of type I IFN-inducing RNAs.

### Mutations slowing ns polyprotein processing increase rPAMP production by replicases of CHIKV and BFV

**Fig 4:**
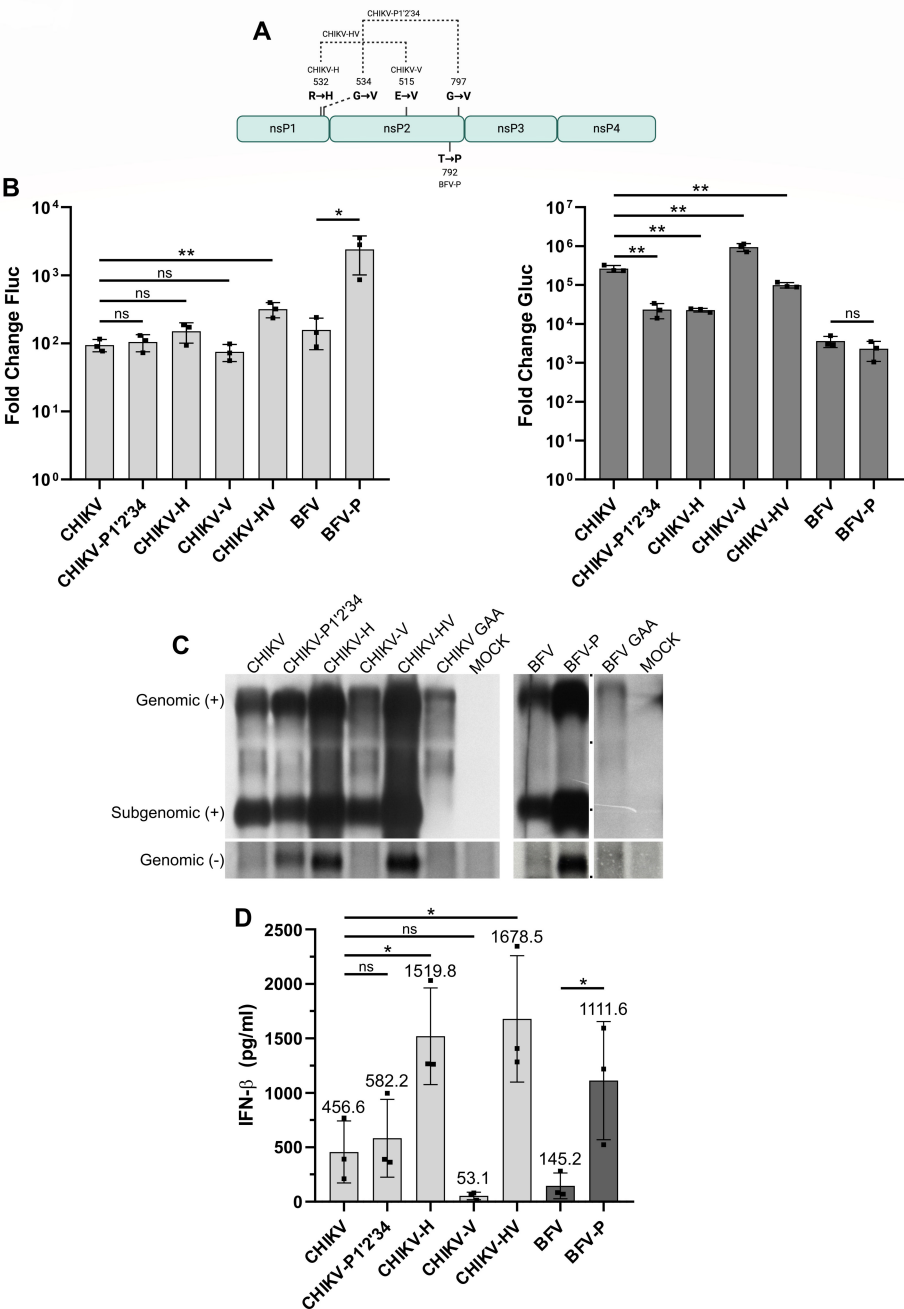
Mutations slowing down CHIKV and BFV ns polyprotein processing increase the production of rPAMPs. (**A**) Schematic representation of CHIKV and BFV ns polyproteins. Point mutations introduced into CHIKV replicase constructs are indicated above the drawing, and the point mutation introduced into BFV replicase is shown below the drawing. The image was created using BioRender. (**B**) HEK293T cells grown on 24-well plates were co-transfected with 0.5 µg HSPolI-FG-CHIKV and 0.5 µg of the indicated CHIKV P1234 expression plasmids or with 0.5 µg HSPolI-FG-BFV and 0.5 µg of the indicated BFV P1234 expression plasmids. In control cells, the CMV-P1234^GAA^-CHIKV or CMV-P1234^GAA^-BFV was used instead of plasmids expressing active replicase. Data were collected, analyzed, and presented as described for Fig. 1D. Statistical analysis was performed using Student’s unpaired *t*-test; **P < 0.01; *P < 0.05; ns, not significant. (**C**) HEK293T cells grown on six-well plates were either mock transfected or co-transfected using 2 µg of the indicated CMV-P1234-CHIKV plasmids and 2 µg HSPolI-FG-CHIKV or 2 µg of the indicated CMV-P1234-BFV plasmids and 2 µg HSPolI-FG-BFV. Cells were collected, analyzed, and data are presented as described for Fig. 1E. (**D**) HEK293T cells grown on 24-well plates were transfected with 2 µg of CMV-P1234 of the indicated CHIKV or BFV variants; control cells were transfected with CMV-P1234^GAA^-CHIKV or with CMV-P1234^GAA^-BFV. The experiment was performed as shown in Fig. 2A, and the data were analyzed and presented as described for Fig. 2B. Statistical analysis was performed using one-way ANOVA; *P < 0.05; ns, not significant.

Previously, we observed that CHIKV infection in mouse tail fibroblasts and *in vivo* was attenuated by substituting the arginine residue at the P4 position of the 1/2 site with histidine and by a glutamic acid-to-valine substitution in the S4 subsite of the nsP2 protease (Fig. 4A; note that the same combination of mutations is present in A774-HV). The mutation in the 1/2 site alone caused similar effects. With both mutations, the attenuation correlated with induction of elevated levels of IFN-α and IFN-β (35), indicating a link between slower P123 processing and increased IFN production.

Here, we found that the *trans*-replicase of CHIKV harboring a substitution either in the 1/2 site or in nsP2 had replication activities similar to those of the wild type (wt) CHIKV replicase. At the same time, a substitution in the 1/2 site significantly decreased transcription activity of CHIKV *trans-*replicase, while the substitution in nsP2 had an opposite effect. When these two substitutions were combined, both replication and transcription activities of the CHIKV *trans*-replicase were slightly but significantly increased. CHIKV replicase harboring mutations completely blocking P123 processing had replication activity similar to that of wt replicase, but its transcription activity was reduced (Fig. 4B). Expectedly, synthesis of viral RNAs measured by northern blot revealed that all mutations that slowed or blocked processing of P123 elevated synthesis of negative-strand RNAs (Fig. 4C). A mutation in the 1/2 site, alone or in combination with a mutation in the nsP2 protease, also increased positive-strand RNA synthesis (Fig. 4C). Thus, the levels of positive-strand RNA synthesis correlated with a boost in Fluc expression (compare Fig. 4B [left panel] and 4C). The increased boost of Gluc expression for CHIKV-V and decreased boost for CHIKV-H did not, however, correlate with observed levels of subgenomic RNAs (compare Fig. 4B [right panel] and 4C). Most likely, this discrepancy was caused by different efficiencies of subgenomic RNA translation that were, apparently, elevated for the case of CHIKV-V replicase and decreased for CHIKV-H replicase. This was not unexpected as the translation of alphavirus subgenomic RNAs depends on the induction of host cell shutdown (36), which, in turn, is affected by mutations in the P123 region (37).

Analysis of viral RNA synthesis by northern blot confirmed that mutations causing a slowdown of processing at the 1/2 site affected the ability of replicases of CHIKV and SFV A774 to synthesize viral RNAs in the same manner (compare Fig. 3C and 4C). Coherently, it was observed that, similar to the case of SFV A774, the substitution in the 1/2 site of the CHIKV ns polyprotein resulted in a prominent and significant increase of rPAMP synthesis (compare Fig. 3D and 4D). In contrast, the complete block of P123 processing had only a minor effect on rPAMP synthesis, and a mutation in the S4 subsite of nsP2 almost completely eliminated this ability (Fig. 4D). Interestingly, levels of rPAMPs produced by the replicase of A774-HV were similar to those produced by the replicase of CHIKV-HV (compare Fig. 3D and 4D). These data confirm that mutations altering the processing of P123 affected viral RNA and rPAMP synthesis of SFV and CHIKV in a similar manner.

Recently, we have found that substitution of the P7 residue of the 2/3 site of BFV from threonine to proline (Fig. 4A) also leads to a slowdown of ns polyprotein processing but increases the production of rPAMPs. Similar to the mutants of RRV and CHIKV, this mutation resulted in diminished replication of BFV in type I IFN-competent mouse embryonic fibroblasts (MEFs) (25). Here, we confirmed that the mutation significantly elevated the replication activity of BFV *trans*-replicase (Fig. 4B), which correlated with increased synthesis of all types of viral RNAs (Fig. 4C). As expected, RNAs isolated from HEK293T cells expressing mutant BFV-P replicase also induced significantly higher levels of IFN-β than did RNAs isolated from cells expressing wt BFV replicase (Fig. 4D). Thus, similar to the case of CHIKV, the reduced replication of mutant BFV in wt MEFs was associated with elevated synthesis of viral RNA and rPAMPs. Interestingly, in contrast to the mutations introduced into SFV and CHIKV replicases, the mutation in P7 position of 2/3 site of BFV had no detectable impact on the 1/2 site processing; instead, it stabilized ns polyproteins by slowing down cleavage of 2/3 site (25). Thus, the speed of processing of the 2/3 site also contributed to the ability of alphavirus replicases to produce rPAMPs. Furthermore, as the processing of the 2/3 site depends on preceding cleavage of the 1/2 site (38) and has been shown to have a critical role in the formation of alphavirus replicase complexes (39), it may represent the key determinant of the ability of alphavirus replicases to synthesize rPAMPs.

### Next-generation sequencing of RNAs from cells expressing SFV replicase or infected by SFV reveals cellular RNAs of opposite polarity

**Fig 5:**
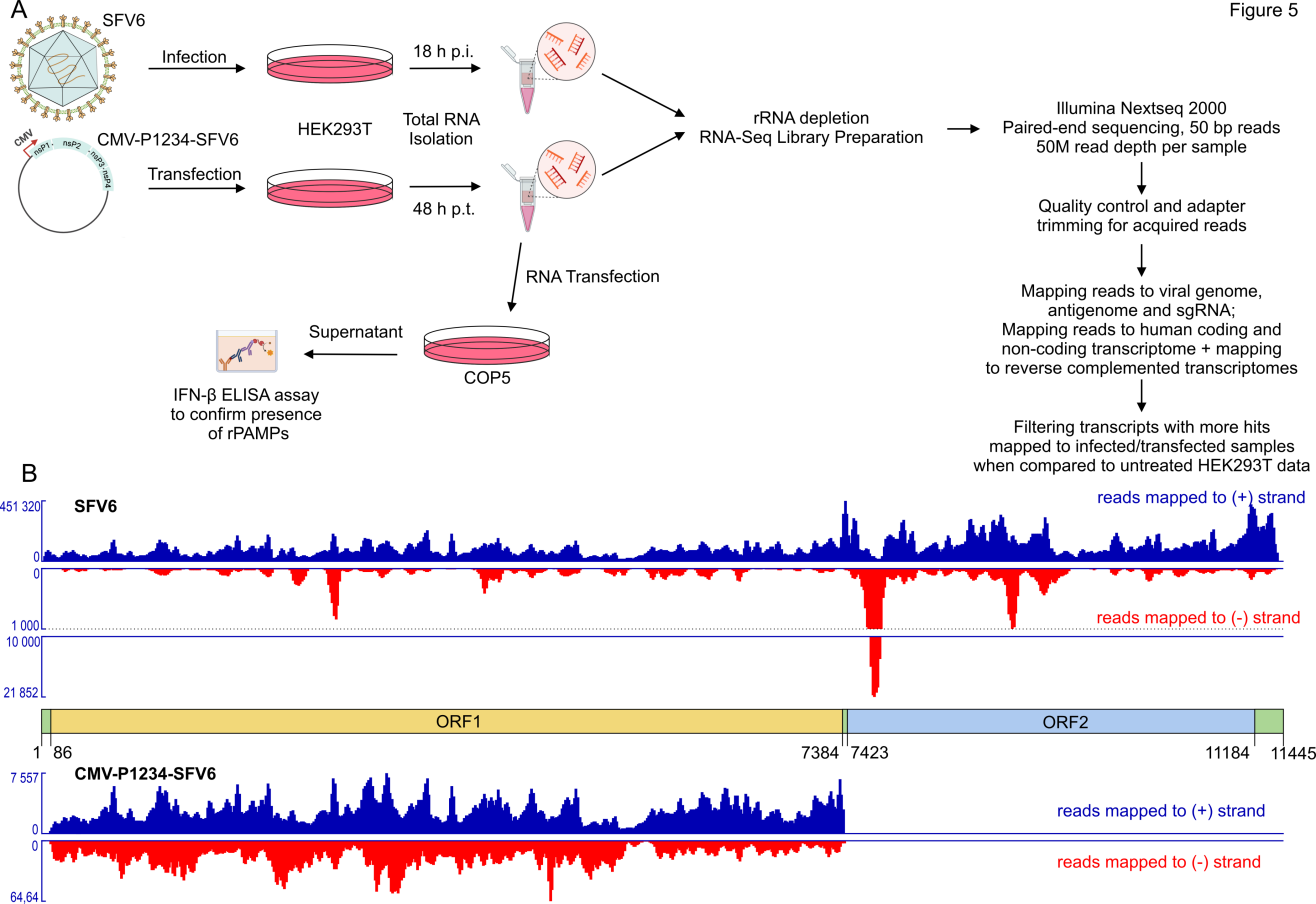
NGS analysis of RNAs isolated from SFV-infected and CMV-P1234-SFV-transfected HEK293T cells. (**A**) Schematic representation of the workflow used to characterize replicase-generated RNAs. HEK293T cells were either infected with SFV6 or transfected with CMV-P1234-SFV6. At the indicated timepoints, cells were collected, and total RNA was isolated. Five micrograms (5 µg) of each total RNA sample was used to transfect COP5 cells to confirm the presence of PAMPs; the remaining RNA samples were used for RNA-seq library preparation. Sequencing was performed using Illumina NextSeq 2000 paired-end sequencing, and the obtained sequences were analyzed as illustrated. The image was created using assets from BioRender. (**B**) RNA-seq read coverage plots showing (top panel) distribution of reads from SFV6-infected cells mapping to SFV genomic RNA (dark blue) and its complementary negative-strand RNA (red). (bottom panel) Distribution of reads from CMV-P1234-SFV6-transfected cells mapping to SFV ORF1 in the sense orientation (dark blue) and antisense orientation (red). The schematic between the panels illustrates the genome organization of SFV6 with respect to the coverage plots’ x-axes, where non-translated regions are shown in green, ORF1 in light brown, and ORF2 in light blue.

rPAMPs are generated by alphavirus replicases using cellular RNA templates. However, it is not known whether they represent homogeneous (generated on a single type of cellular RNA template) or heterogeneous (generated on multiple types of cellular RNA templates) dsRNA populations. Here, we attempted to address this question first using SFV6 infection/SFV6-P1234 expression combined with a standard next-generation sequencing (NGS) workflow (Fig. 5A). Total RNAs were isolated from HEK293T cells transfected using the SFV6 replicase expression plasmid or infected with SFV6. Cell culture supernatant of COP5 cells transfected using 5 μg of total RNA isolated from HEK293T cells expressing SFV6 replicase contained ~1,500 pg/mL of IFN-β, an amount that is consistent with that detected in other experiments (Fig. 2B and 3D). 5 μg of RNA isolated from SFV-infected HEK293T cells induced marginally higher (~1,700 pg/mL) amounts of IFN-β. These data confirm high abundance and/or potency of PAMPs present in the isolated RNA samples.

The isolated total RNA samples were depleted of rRNA and used for strand-specific library preparation for 50 bp paired-end read sequencing on the Illumina platform. For RNAs isolated from SFV6-infected cells, we obtained ~66 million reads. 29.9 million reads matched to the positive-strand RNA of SFV6. The sequence coverage of the region corresponding to the subgenomic RNA (ORF2 and the 3′UTR) was slightly higher than that of the ORF1 region, which is present only in the genomic RNA. A total of 14.6 million reads matched to the ORF1 region, distributed over the entire region with modest differences in coverage along the sequence (Fig. 5B). Additionally, ~75,000 reads matched the negative-strand RNA of SFV6. The highest sequence coverage by far was observed in the beginning of ORF2, that is, the region corresponding to the beginning of the capsid protein-encoding region (Fig. 5B). As ORF1 comprises ~63.8% from the total length of SFV6 genome, the calculated ratio of negative- to positive-strand genomic RNAs in the sample was approximately 1:300. This is much lower than expected 1: 20 based on reports that during their replication, alphaviruses generate up to 10,000 negative- and 200,000 new genomic RNAs per infected cell (40) and that their dsRNA replication intermediate is an abundant, easily detectable molecule (23). Thus, the low number of negative-strand reads indicates that the majority of viral dsRNAs were lost, presumably during the generation of sequencing libraries that included an rRNA depletion workflow, which relied on hybridization of rRNA and globin mRNA targets and subsequent enzymatic degradation.

Approximately 54 million reads were obtained for RNAs isolated from cells transfected with the SFV6 replicase expression plasmid. Out of these ~534,000 reads matched to the ORF encoding for SFV ns polyprotein. Notably, the sequence coverage along the ORF1 followed a pattern highly similar to that observed for viral positive-strand RNAs from SFV6-infected cells (Fig. 5B). Unexpectedly, we also detected ~3,500 reads matching ORF1 in the negative orientation. These reads were relatively evenly distributed along the ORF1, displaying a pattern clearly different from that observed for negative-strand RNAs from SFV-infected cells (Fig. 5B). Thus, these reads likely had a different origin and were probably generated from transcripts synthesized by cellular RNA polymerases using cryptic promoter(s) in the replicase expression plasmid. Their high relative abundance (negative- to positive-strand ratio 1:150) indicates that the method used is poorly suited for detecting true rPAMPs.

When aligning reads to the human non-coding RNA transcriptome and the corresponding reverse-complemented transcriptome, we did however notice reads mapping to the opposite polarity transcripts of three different long non-coding RNA (lncRNA) genes; these matches included SSTR5 antisense RNA 1 (ENSG00000261713) and two novel transcript genes with little annotation (ENSG00000254859, ENSG00000307362). While samples from replicase-expressing and infected cells aligned with very few reads to the positive-sense lncRNA transcripts (less than 60 reads per transcript), infected sample reads mapped to the opposite polarity transcripts at least 12,000 times, and replicase-expressing sample reads at least 20,000 times. The mapping was also performed using a previously published untreated HEK293T total RNA sequencing data (NCBI SRA: SRX2867694) as a control. For this data set, these lncRNA alignments were both in very low abundance (never reaching numbers over 200 reads per transcript) and were similarly represented for references of either transcript strand polarity. All the transcripts from these genes have been described to be between 400 and 3,000 bases in length, certainly fitting the previous experimental rPAMP descriptions (23); nonetheless, we cannot decidedly conclude with our current data that these transcripts are rPAMPs or that the RNAs from detected genes would represent rPAMP templates, as a higher number of samples would be necessary to assume any statistical correlation. Previously, RNA antisense to the mouse non-coding mitochondrial RNA 1 ASncmtRNA-1 (GenBank: GU332589.1) has been detected as a potential rPAMP in murine cells (23). Therefore, we specifically looked for reads antisense to the human ortholog, the non-coding mitochondrial RNA 2 ASncmtRNA-2 (GenBank: EU863790.1). Such reads were present in both analyzed RNA samples; however, their abundance was similar to that observed for samples from non-treated HEK293T cells. Thus, these reads represented a consequence of the expression strategy of these cellular RNAs, and replicase-generated rPAMPs, if present, could not be detected. Given the fact that libraries prepared using RNAs from SFV6-infected cells were strongly depleted of viral negative-strand RNAs, it is likely that such dsRNAs, generated by viral replicase, were lost during the preparation of sequencing libraries.

### rPAMPs can be pulled down using Flag-tagged RIG-I

**Fig 6:**
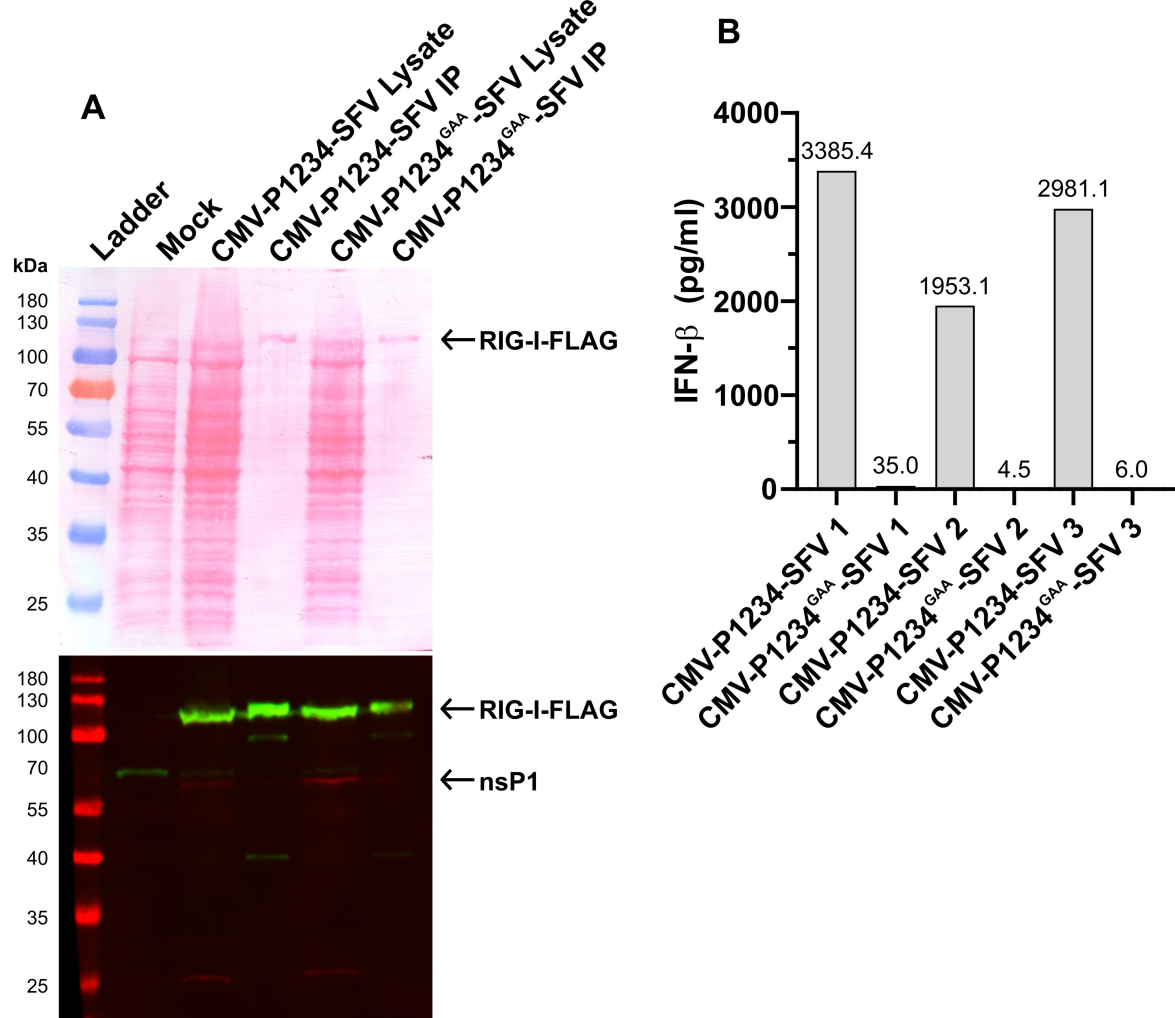
RIG-I pull-down of rPAMPs. HEK293T cells were first transfected with pcDNA4/TO-RIG-I-Flag and, after 12 h, transfected with either CMV-P1234-SFV or CMV-P1234-SFV^GAA^, followed by incubation for 24 h. Cells were harvested, lysed, and RIG-I-mediated rPAMP pull-down was performed via the Flag tag. (**A**) SDS-PAGE analysis of input (Lysate) and pull-down (IP) fractions visualized by Ponceau S staining (upper panel); immunoblot detection of RIG-I-Flag and SFV nsP1 in lysate and IP fractions using antibodies against the Flag tag and SFV nsP1 (lower panel). Three independent experiments produced similar results; data from one representative experiment are shown. (**B**) COP5 cells were transfected with RNAs pulled down with RIG-I-Flag to confirm the presence of rPAMPs. Induced IFN-β levels are shown for each sample that was subsequently used for NGS analysis.

RIG-I is among the primary cellular PRRs sensing rPAMPs (23). To confirm and exploit this interaction, HEK293T cells were co-transfected with an expression plasmid encoding C-terminally Flag-tagged RIG-I (RIG-I-Flag) together with either CMV-P1234-SFV or its polymerase-deficient mutant, CMV-P1234-SFV^GAA^, and subjected to Flag-based immunoprecipitation. SDS-PAGE and immunoblot analyses confirmed the successful pull-down of RIG-I (Fig. 6A). Transfection of the RNAs co-purified with RIG-I-Flag into COP5 cells resulted in robust IFN-β induction from samples derived from cells expressing the active SFV replicase but not from those expressing the polymerase-negative variant (Fig. 6B). These results confirm that polymerase activity is required for the synthesis of rPAMPs and that these RNAs directly associate with RIG-I.

The isolated rPAMP samples were next subjected to Illumina stranded RNA sequencing, where the library preparation method remained the same as described above, but here the rRNA depletion step was omitted. Although the expected number of reads (~80–100 million read pairs) was obtained for all samples, approximately 90% of reads were classified as PCR duplicates, indicating low library complexity. Consequently, comparative analysis of the CMV-P1234-SFV and CMV-P1234-SFV^GAA^ datasets did not reveal any distinct or highly abundant non-canonical RNA species attributable to replicase activity. To address potential limitations associated with short-read library preparation, the rPAMP pull-down samples were also analyzed using a long-read Oxford Nanopore Technologies (ONT) sequencing platform. Because rPAMPs lack poly(A) tails (23), a cDNA-based library preparation approach was employed. However, the ONT cDNA sequencing runs yielded data of insufficient quality, with the majority of reads (~98%) being shorter than 200 nt and approximately 83% of reads failing to map to human sequences.

Collectively, these observations indicate that standard NGS library preparation workflows are not suitable for the analysis of rPAMPs. The likely cause is inefficient cDNA synthesis from dsRNA molecules longer than ~200 bp (23), resulting in poor library complexity and the absence of abundant antisense reads corresponding to host or viral transcripts. Therefore, conventional short- and long-read sequencing methods currently do not provide reliable means for comprehensive rPAMP characterization.

### Replicases of several alphaviruses synthesize type I IFN-inducing RNAs in mosquito cells

**Fig 7:**
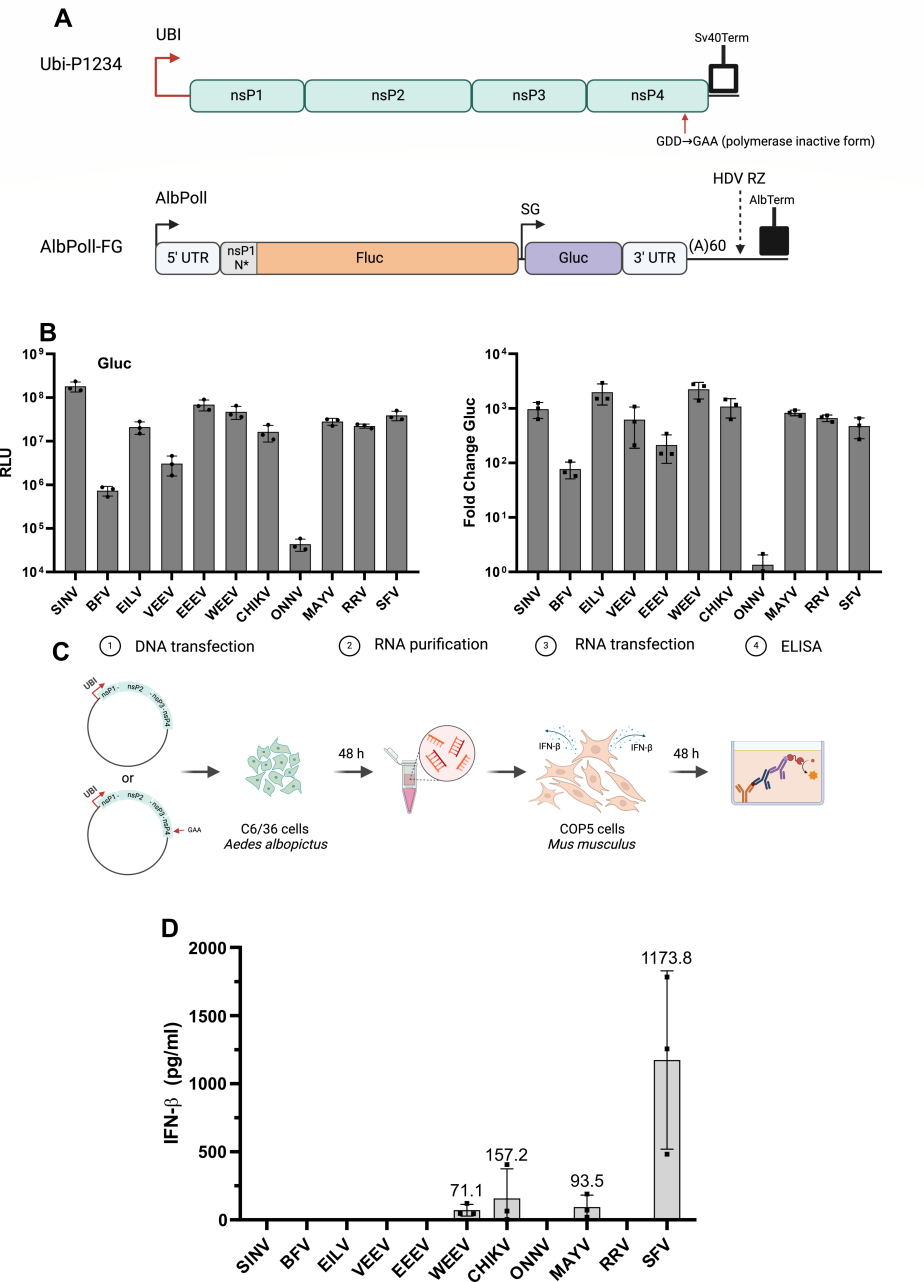
IFN-inducing RNAs are also synthesized in mosquito cells. (**A**) Schematic presentation of expression constructs for alphavirus replicase and mini-genome for *Aedes albopictus* cells. Ubi, promoter for polyubiquitin gene in *Aedes aegypti*; AlbPolI, truncated promoter for RNA polymerase I of *Aedes albopictus*; AlbTerm, a terminator for RNA polymerase I of *Aedes albopictus*. Other designations are the same as on Fig. 1A. The image was created using BioRender. (**B**) C6/36 cells grown on 24-well plates were co-transfected with 0.5 µg of Ubi-P1234 and 0.5 µg of corresponding AlbPolI-FG plasmids; for control cells, Ubi-P1234^GAA^ was used instead of the plasmid expressing active replicase. Cells were lysed at 48 hpt, and the activities of Gluc were measured. (Left panel) Absolute Gluc activities presented in RLU per 20,000 transfected cells. (Right panel) Activities of Gluc in cells expressing active replicases were normalized to those in control cells (taken as 1). Individual data points, mean values, and SD from three independent experiments are shown. (**C**) Schema of experiment used for the detection of type I IFN-inducing RNAs produced in C6/36 cells. Cells grown on 24-well plates were transfected with 2 µg of Ubi-P1234 or Ubi-P1234^GAA^ of each analyzed virus. At 48 hpt, cells were collected, and total RNA was isolated. 5 µg of total RNA was used to transfect COP5 cells, and amounts of secreted IFN-β-cell culture medium were measured at 48 hpt. Image was created using BioRender. (**D**) Induction of IFN-β by RNAs isolated from C6/36 cells transfected with expression plasmids of replicases of indicated alphaviruses. Data were analyzed and are presented as described for Fig. 2B.

Though mosquitoes lack type I IFN system, they do use dsRNAs for pathogen detection (41) as well as for the generation of exogenous siRNAs (42). To analyze the synthesis of type I IFN-inducing RNAs in transfected mosquito cells, we exploited alphavirus replicase expression plasmids and *trans*-replicases developed for *Aedes albopictus* cells (Fig. 7A). In C6/36 cells, *trans*-replicase of ONNV was nearly inactive; those of BFV and VEEV were also considerably less active than others (Fig. 7B). This is consistent with our previous observations and may reflect the fact that these alphaviruses are not transmitted by *Aedes albopictus* mosquitoes (30). *Trans-*replicases of EEEV and WEEV, which have previously not been characterized in mosquito cells, were both capable of induction of high levels of Gluc expression (Fig. 7B, left panel); for replicase of EEEV, the normalized boost of Gluc signal was somewhat diminished (Fig. 7B, right panel) due to higher background Gluc expression from the EEEV template RNA.

Next, we adopted our approach for the detection of type I IFN-inducing RNAs produced in cells transfected with replicase expression plasmids for C6/36 cells (Fig. 7C). The analysis revealed that RNAs isolated from C6/36 cells expressing replicase of SFV induced very high levels of IFN-β expression in transfected COP5 cells. The potency of RNAs isolated from C6/36 cells expressing replicases of other alphaviruses to induce IFN-β expression in transfected COP5 was much lower or undetectable (Fig. 7D). In part, this may reflect lower transfection efficiency of C6/36 cells (compared to HEK293T cells), which diminishes replicase expression and drastically reduces the numbers of cells where viral RNA synthesis occurs (30). Interestingly, no clear correlation between the synthesis of type I IFN-inducing RNAs (Fig. 7D), the activities of *trans-*replicases (Fig. 7B), or their previously reported abilities to synthesize viral positive-strand RNAs (30) was observed. Other than SFV replicase, only replicases of MAYV, CHIKV, and WEEV produced detectable levels of type I IFN-inducing RNAs in C6/36 cells (Fig. 7D). For replicases of ONNV, BFV, and VEEV, the lack of synthesis of type I IFN-inducing RNAs could be a consequence of their low activities in C6/36 cells (Fig. 7B). However, this is unlikely the only or the main reason as *trans-*replicases of SINV, EILV, EEEV, and RRV were highly active in C6/36 cells (Fig. 7B) yet failed to synthesize type I IFN-inducing RNAs (Fig. 7D).

## DISCUSSION

The ability to use cellular RNA templates for the synthesis of RNAs that trigger type I IFN response was first described for the replicase of SFV and then for replicases of SINV, RRV, and BFV (23–25). Here, we demonstrate that replicases of 11 alphaviruses, representing six antigenic complexes, share this ability.

In human cells, the abilities of alphavirus replicases to replicate corresponding template RNAs and to synthesize rPAMPs generally correlate with each other. As rPAMPs are dsRNAs, it could be assumed that they are produced by the P123 + nsP4 complex, the early replicase that synthesizes negative strands of viral dsRNA replication intermediate. Coherent with this correlation, mutations that stabilize the early replicase complex increase rPAMP production by replicases of SFV, CHIKV, and BFV (Fig. 3D and 4D) as well as synthesis of viral negative-strand RNAs; similarly, the slowdown of P123 processing has also been shown to increase negative-strand RNA synthesis and rPAMP production by RRV (24). Interestingly, however, CHIKV replicase containing uncleavable P123 synthesizes elevated levels of viral negative-strand RNAs but not rPAMPs (Fig. 4C and D). Therefore, it is plausible that slow-down P123 processing facilitates the formation of increased amounts of complexes capable of rPAMP synthesis but does not increase their activity. The nature of these complexes remains unknown. It is unlikely that rPAMPs are products of classical alphavirus replicase complexes where the nsP4 has only access to the dsRNA present inside the spherule (10). To perform the synthesis of rPAMPs, spherules should contain heterologous dsRNA molecules that are considerably shorter than viral replication intermediates (23). As the size of spherules depends on the length of the dsRNA (43), the use of shorter RNAs should result in the formation of spherules with considerably smaller sizes; to the best of our knowledge, this has not yet been documented. Furthermore, rPAMPs are made by the cleavage products of wt ns polyproteins in the absence of template RNA, that is, at conditions where spherules are not formed (39). On the other hand, individual recombinant ns proteins of alphaviruses assemble into complexes similar to the replicase cores capable of limited extension of provided non-viral template RNAs. Interestingly, similar complexes are abundant in alphavirus-infected cells (10), making it plausible that these structures, or possibly their precursors, may be associated with rPAMP synthesis.

Synthesis of rPAMPs appears to be a common feature of alphavirus replicases, suggesting that these molecules are either functionally important or simply by-products of highly efficient replicase activity that spills over onto host cell RNAs. Their production in mosquito cells, where these replicase products are presumably inactive, is consistent with the latter possibility. However, the finding that replicases of different alphaviruses—and, in some cases, different strains and mutants—synthesize markedly different amounts of rPAMPs (Fig. 2B), together with reported phenotypic differences among these viruses, argues that rPAMPs may be biologically relevant and that their functional significance may vary among viruses.

SINV and SFV are the most efficient rPAMP producers (Fig. 2B), and the elevated rPAMP synthesis—along with the corresponding increase in type I IFN induction resulting from increased stability of P123—does not appear to be harmful to these viruses. It is possible that the negative impact of increased type I IFN induction is compensated for by more efficient virus replication. SFV and SINV also share several similarities: they both have naturally occurring strains with elevated rPAMP production, and they both cause lethal encephalitis in mouse models. It has been reported that more severe outcomes of SINV encephalitis are linked to neuronal apoptosis (44) and that SFV replication, resulting in dsRNA synthesis, stimulates an apoptotic pathway via MDA5 activation (45). Therefore, it is conceivable that rPAMPs could play a role in these processes. Synthesis of these molecules may, directly or via induction of excessive amounts of type I IFN, increase the susceptibility of cells of the central nervous system for the infection by SFV or SINV. Interestingly, however, replicases of encephalitic New World alphaviruses synthesize only modest/low levels of rPAMPs (Fig. 2B). Individual virus antagonism of type I IFN induction is a major contributor to virulence and could mask *in vitro* induction phenotypes. It is therefore possible that due to significant differences in virus-host interactions, these viruses do not require rPAMPs to infect the central nervous system.

The data obtained for RRV, CHIKV, and BFV are seemingly consistent with the hypothesis that the synthesis of rPAMPs can represent unwanted consequences of high *trans*-activity of alphavirus RNA replicase and efficient viral RNA synthesis. A slowdown of P123 processing reduces RRV replication in wt MEFs and reduces virus pathogenicity in wt mice (24); the same is the case for CHIKV (35). These differences from SFV may be due to the fact that RRV and CHIKV, replicases of which generate much lower levels of rPAMPs (Fig. 2B), are not adapted to the use of large amounts of these molecules for benefits of infection. Furthermore, in contrast to the cases of SINV and SFV strains, the mutations slowing down ns polyprotein processing of CHIKV, RRV, and BFV are artificial. Therefore, for these viruses, the increased rPAMP synthesis represents an artificially generated phenomenon, making it likely that the viruses are unable to use it for their benefit and become attenuated instead. However, the inability to use excessive amounts of rPAMPs to enhance the *in vivo* infection does not mean that these molecules are useless or outright harmful. rPAMPs are at least as important contributors to the type I IFN induction as are more classical virus-generated PAMP RNAs (24). While the activation of anti-viral immune responses may seem counterproductive for virus infection, it may actually contribute to virus survival. Thus, a very recent study revealed that activation of type I IFN response restricts infection of DCs; this is favorable for alphavirus as it reduces direct antigen presentation and anti-viral CD8 T-cell response, preventing clearance of virus-infected cells (22). Taken together, it is likely that even seemingly harmful by-products of RNA replicase actually contribute to the important properties of alphaviruses, including their tissue tropism, pathogenesis, and persistence.

Unexpectedly, it was found that the replicase of SFV, and to some extent those of CHIKV, MAYV, and WEEV, can also generate rPAMPs in mosquito cells. Except for WEEV, all these viruses belong to the Semliki Forest virus complex. Replicases of viruses from this complex have the ability to copy heterologous template RNAs (30). Hence, it can be speculated that in mosquito cells, this property may be a prerequisite for rPAMP synthesis. Consistent with this hypothesis, the replicase of SINV that produces high levels of rPAMPs in human cells failed to do this in mosquito cells. It has been previously shown that in C6/36 cells, SINV replicase uses efficiently only matching template RNA (30), probably because the structural elements in SINV template RNA, essential to its functioning, are rather virus-specific (46). Thus, more strict template RNA requirements of SINV replicase may prevent the use of cellular RNAs from mosquito cells.

rPAMPs are typically dsRNA molecules longer than 200 bp, lack poly(A) tails, and are primarily recognized by RIG-I (23). Co-immunoprecipitation approaches have been successfully used to characterize RIG-I- and MDA5-bound RNA ligands produced during Sendai virus (47) and measles virus (48) infections. A similar strategy applied to cells expressing the SFV6 replicase was successfully implemented in this study (Fig. 6). However, our repeated attempts to characterize rPAMPs using NGS libraries prepared from either total RNA or from RNAs pulled down via RIG-I did not result in conclusive identification of these molecules. Given that these RNA samples were highly potent inducers of IFN-β expression (Fig. 6B), the outcome most likely reflects technical limitations of sequencing library preparation. Consistent issues observed across two different sequencing platforms suggest a common difficulty in capturing dsRNA for efficient cDNA synthesis and incorporation into NGS libraries. Similar difficulties have been reported for recovering genomic reads of the dsRNA bacteriophage φ6 from defined microbial communities using standard Illumina library preparation methods (49). Inclusion of DMSO during heat denaturation has been shown to increase sequencing yield for phage φ6 genomes by more than two orders of magnitude (50) and has also been successfully applied in metagenomic studies for the discovery of novel dsRNA viruses (51). Hence, future efforts to elucidate the molecular composition of alphavirus rPAMPs should employ library preparation protocols specifically optimized for dsRNA, such as DMSO-assisted dsRNA denaturation, to improve sequencing library generation. Finally, our inability to identify cellular RNAs that serve as templates for rPAMP formation may also suggest that these molecules are generated *de novo* by alphavirus replicase. Alphaviruses have been shown to produce defective interfering RNAs containing short fragments of cellular tRNA sequences (52, 53). Moreover, the alphavirus replicase can add adenosine or uridine residues in a template-independent manner (54, 55). If so, rPAMPs may be intrinsically heterogeneous and lack extensive sequence similarity to canonical host or viral RNAs, complicating their distinction from background artifacts introduced during library preparation and sequencing. Although no direct evidence currently supports this possibility, it cannot be entirely excluded.

Linking *in vitro* findings to *in vivo* phenotypes remains challenging. In mammalian hosts, alphaviruses infect and replicate in multiple cell types, including fibroblasts, dendritic cells, macrophages, muscle cells, and, in some cases, neurons; however, rPAMP synthesis in these specific cell types remains unknown. It is likely that cell type–specific responses contribute to *in vivo* outcomes, and differences in tissue tropism may help explain the distinct phenotypes previously observed for SFV, RRV, and CHIKV. It cannot be excluded that rPAMPs produced in mosquito cells are simply by-products of viral replicase activity. Nevertheless, because *in vivo* phenotypes of alphaviruses in their vertebrate hosts correlate with rPAMP production, these RNAs may also have functional significance during infection of mosquito vectors; dedicated studies will be required to determine whether, and how, rPAMPs influence alphavirus infection in mosquitoes.

Beyond understanding mechanisms of viral pathogenesis, our findings have practical implications for alphavirus-based biotechnology. Alphaviruses are the focus of intense vaccine development efforts, with chikungunya virus leading the field due to its global disease burden and pandemic potential (56). Alphavirus-based self-amplifying RNA (saRNA) vaccine candidates have entered clinical trials (57, 58), demonstrating the feasibility of this platform for advanced vaccine development, and the *trans*-activity of alphavirus replicases has been exploited for the development of *trans*-amplifying RNA (taRNA) technology (59). While non-replicating mRNA vaccines exhibit beneficial self-adjuvant effects primarily through endosomal sensing of RNA backbones (60), saRNA and taRNA platforms generate dsRNAs, including rPAMPs, and are therefore expected to elicit stronger innate immune responses. However, strong saRNA-induced type I IFN responses have been shown to reduce the production of the desired antigen, and delayed processing of the 1/2 site results in impaired antigen expression in mouse models (61). A better understanding of the mechanisms by which alphavirus-based replicating RNAs induce type I IFN responses therefore offers opportunities to regulate immunogenicity by rationally tuning replicase activity to produce appropriate amounts of rPAMPs. Moreover, the use of replicases from alphaviruses that not only synthesize rPAMPs but may also benefit from their production represents an attractive strategy. Together, these rational approaches could combine beneficial self-adjuvant properties with efficient antigen expression and thereby improve the performance of saRNA- and taRNA-based vaccine candidates.

## MATERIALS AND METHODS

### Cell lines and viruses

HEK293T cells (ATCC, CRL-3216) were cultured in Dulbecco’s modified Eagle’s medium (DMEM, Corning) supplemented with 10% fetal calf serum (FCS, Pan Biotech). COP5 cells (62) were cultured in Iscove’s modified DMEM (IMDM, Corning) supplemented with 10% FCS. Baby hamster kidney cells (BHK-21; ATCC CCL-10) were maintained in Glasgow’s modified Eagle’s medium (GMEM) supplemented with 10% FBS, 20 mM HEPES, 10% tryptose phosphate broth (TPB), and 1 mM l-glutamine. *Aedes albopictus* C6/36 cells (ATCC, CRL-1660) were maintained in Leibowitz’s L-15 medium (Corning) supplemented with 10% FCS and 10% tryptose phosphate broth (Gibco). HEK293T, COP5, and BHK-21 cells were grown in a humidified incubator at 37°C with 5% CO_2_. C6/36 cells were grown in a humidified incubator at 28 °C without the addition of CO_2_.

SFV6 was rescued from the corresponding icDNA clone. Briefly, BHK-21 cells were transfected using 2 μg of plasmid containing icDNA of SFV6 (34) and cells were incubated at 37 °C for 24 h. Cell culture supernatant was clarified by centrifugation at 1,000 × *g* for 10 min, filtered through 0.22 μm filter, titered using plaque titration on BHK-21 cells, and stored in aliquots at −80°C.

### Plasmids

Human RNA polymerase I promoter-based expression plasmids designed to produce amplification-competent template RNAs designated HSPolI-FG-SFV, HSPolI-FG-EEEV, HSPolI-FG-MAYV, HSPolI-FG-SINV, HSPolI-FG-BFV, HSPolI-FG-RRV, HSPolI-FG-VEEV, HSPolI-FG-CHIKV, HSPolI-FG-ONNV, and HSPolI-FG-EILV have been previously described (30, 31, 63–65). The template RNA-expressing plasmid designated HSPolI-FG-WEEV had a similar design. Plasmids expressing P1234 alphaviruses in mammalian cells, designated as CMV-P1234-SFV, CMV-P1234-EEEV, CMV-P1234-MAYV, CMV-P1234-SINV, CMV-P1234-BFV, CMV-P1234-RRV, CMV-P1234-VEEV, CMV-P1234-CHIKV, CMV-P1234-ONNV, and CMV-P1234-EILV and their variants encoding polymerase-negative variant P1234^GAA^ (CMV-P1234^GAA^-SFV and so on) have also been previously described (31). Expression plasmids of P1234 and P1234^GAA^ of WEEV were constructed in the same way and designated CMV-P1234-WEEV and CMV-P1234^GAA^-WEEV, respectively. Plasmids CMV-P1234-SFV-A774 and CMV-P1234-SFV-A774-HV are described in reference 65; plasmids CMV-P1^GV^2^GV^34-CHIKV, CMV-P1^RH^234-CHIKV, CMV-P12^EV^34-CHIKV, and CMV-P1^RH^2^EV^34-CHIKV are described in reference 65, and plasmid CMV-P12^TP^34-BFV is described in reference 25. Plasmid CMV-P1234-SFV6-74-RE was constructed from pCMV-SFV6-74-RE (34) and CMV-P1234-SFV using restriction enzyme-based cloning.

*Aedes albopictus* RNA polymerase I promoter-based expression plasmids for expression of amplification-competent template RNAs designated AlbPolI-FG-SFV, AlbPolI-FG-MAYV, AlbPolI-FG-SINV, AlbPolI-FG-BFV, AlbPolI-FG-RRV, AlbPolI-FG-VEEV, AlbPolI-FG-CHIKV, AlbPolI-FG-ONNV, and AlbPolI-FG-EILV have been previously described (30, 31, 63–65). Template RNA-expressing plasmids named AlbPolI-FG-EEEV and AlbPolI-FG-WEEV had similar design. Plasmids expressing P1234 of nine alphaviruses in C6/36 cells, designated Ubi-P1234-SFV, Ubi-P1234-MAYV, Ubi-P1234-SINV, Ubi-P1234-BFV, Ubi-P1234-RRV, Ubi-P1234-VEEV, Ubi-P1234-CHIKV, Ubi-P1234-ONNV, and Ubi-P1234-EILV and their variants encoding polymerase-negative variant P1234^GAA^ (Ubi-P1234^GAA^-SFV and so on) have been previously described (30). Plasmid expressing P1234 and P1234^GAA^ of EEEV and WEEV in C6/36 cells was constructed in the same way and designated Ubi-P1234-EEEV, Ubi-P1234-WEEV, Ubi-P1234^GAA^-EEEV, and Ubi-P1234^GAA^-WEEV. All new P1234 and template RNA expression plasmids were constructed using synthetic DNA fragments (Genscript), site-directed mutagenesis, and restriction enzyme-based cloning procedures; their sequences were verified using Sanger sequencing. The sequence encoding human RIG-I with a C-terminal Flag tag was obtained from a pre-existing pEF-BOS backbone (66) based expression construct and cloned into the pcDNA4/TO (Invitrogen) vector by restriction enzyme cloning, verified by sequencing, and designated as pcDNA4/TO-RIG-I-Flag.

### Trans-replication assays

The *trans*-replicase assays with plasmids that express template RNAs using RNA polymerase I promoters were performed as previously described (63). Briefly, HEK293T cells in 24-well plates were grown to ~80% confluence and then transfected with 0.5 μg of replicase expression plasmids (CMV-P1234 or its mutant variants) and 0.5 μg of matching template RNA plasmids (HSPolI-FG) using Lipofectamine LTX with PLUS reagent according to the manufacturer’s instructions. In the control cells, the replicase-expressing plasmid was replaced with the expression plasmid of P1234^GAA^. The transfected cells were incubated at 37°C for 18 h. The same procedure was used for C6/36 cells, except using replicases (Ubi-P1234) and template RNA (AlbPol-FG) expression plasmids designed for use in mosquito cells, and that transfected cells were incubated at 28°C for 48 h. After incubation, the cells were collected, lysed using lysis buffer (Promega), and Fluc and Gluc activities were measured using a dual-luciferase reporter kit and GloMAX SIS luminometer (Promega). Fluc and Gluc activities from each experiment were normalized to those obtained from matching control cells (corresponding values were taken as 1).

To analyze expression kinetics of Gluc marker, COP5 cells were grown in 24-well plates to a ~80%–90% confluency and co-transfected with 0.5 μg of replicase expression plasmids (CMV-P1234) and 0.5 μg of matching template RNA expression plasmids (HSPolI-FG) using Lipofectamine 2000 reagent (Invitrogen) according to the manufacturer’s protocol. At 4, 8, 12, 18, 24, and 48 hpt, 20 µL of cell culture medium was harvested from each well; 4 µL of each obtained sample was used to detect the Gluc activity using Renilla Luciferase Assay System and GloMAX SIS luminometer (Promega).

### rPAMP production and purification

HEK293T cells grown in 24-well plates were transfected with 2 µg of replicase expression plasmids (CMV-P1234 or their mutant variants). Similarly, C6/36 cells grown in 24-well plates were transfected with 2 µg of replicase expression plasmids (Ubi-P1234). In both cases, control cells were transfected with equal amounts of plasmid expressing corresponding polymerase-negative replicase polyprotein (CMV-P1234^GAA^ for human cells and Ubi-P1234^GAA^ for mosquito cells). Transfections were performed using Lipofectamine LTX and PLUS reagent (Invitrogen) according to the manufacturer’s protocol. After 48 h, cells were harvested, and total RNA was purified using TRIzol reagent (Invitrogen) according to the manufacturer’s protocol.

### IFN-β induction and detection

For IFN-β induction, 5 µg of purified total RNA from transfected HEK293T or C6/36 cells was transfected into confluent COP5 cells grown in 24-well plates using the Lipofectamine 2000 reagent (Invitrogen); for each transfection, 5 µg purified RNA was mixed with 5 µL Lipofectamine 2000. After 48 h, secreted IFN-β was detected in the cell culture medium by enzyme-linked immunosorbent assay (ELISA) using the Mouse IFN-Beta ELISA kit (PBL assay, 42400) according to the manufacturer’s protocol.

### Northern blotting

HEK 293T cells grown in six-well plates were co-transfected with 2 µg of replicase expression plasmids (CMV-P1234-WEEV, CMV-P1234^GAA^-WEEV, CMV-SFV-P1234, CMV-P1234-SFV-A774, CMV-P1234-SFV-A774-HV, CMV-P1234-SFV6-74-RE, CMV-P1234^GAA^-SFV, CMV-P1234-CHIKV, CMV-P1^GV^2^GV^34-CHIKV, CMV-P1^RH^234-CHIKV, CMV-P12^EV^34-CHIKV, CMV-P1^RH^2^EV^34-CHIKV, CMV-P1234^GAA^-CHIKV, CMV-P1234-BFV, CMV-P12^TP^34-BFV, or CMV-P1234^GAA^-BFV) and 2 µg of matching template RNA expression plasmid (HSPolI-FG-WEEV, HSPolI-FG-SFV, HSPolI-FG-BFV, or HSPolI-FG-CHIKV). 18 hpt cells were lysed, and total RNA was purified using TRIzol reagent (Invitrogen) according to the manufacturer’s protocol. Northern blotting was performed as previously described (30). Briefly, 2.5 µg of total RNA was used for the detection of positive-strand RNAs, and 10 µg of total RNA was used for detection of negative-strand RNA. RNAs were separated by formaldehyde agarose gel-electrophoresis and transferred to nylon membrane (Amersham Hybond-N+, Cytiva). Positive-strand transcripts were hybridized with digoxigenin-labeled RNA probes complementary to sequence encoding for Gluc marker, and negative-strand transcripts were detected by RNA probes matching to sequence encoding for Fluc marker. RNAs were visualized by chemiluminescence using anti-digoxigenin-AP antibodies, DIG Wash, and Blocking Buffer Set and *CDP*-Star reagent (all from Roche) according to the manufacturer’s instructions.

### NGS analysis of RNAs isolated from replicase-expressing and infected cells

Confluent HEK 293T cells grown on six-well plates were transfected with CMV-SFV-P1234 or infected with SFV6 at an MOI of 5. Cells were lysed at 48 h pt or 18 h post-infection, total RNAs were purified using TRIzol reagent (Invitrogen), and their ability to induce IFN-β induction was confirmed using the above-described protocol. The RNA sequencing libraries were prepared using the Illumina Stranded Total RNA Prep with Ribo-Zero Plus kit and sequenced with the NextSeq2000 P1 chip (Illumina) with 50 bp paired-end reads in the sequencing facility of Institute of Genomics, University of Tartu. Bowtie2 (67) was used for read mapping to reference genomes, SeqKit (68) was used to produce reverse complement reference sequences, and SAMtools (69) was used for file conversions. Read coverage plots were visualized using the Integrative Genomics Viewer (70).

### Pull-down of rPAMPs bound to RIG-I and NGS

HEK293T cells grown in T-75 flasks were transfected with 40 µg of plasmid expressing Flag-tagged RIG-I (pcDNA4/TO-RIG-I-Flag). After incubation at 37°C for 12 h, cells were transfected with either 40 µg of CMV-SFV-P1234 or CMV-SFV-P1234^GAA^. Both transfections were performed using Lipofectamine LTX and PLUS reagent (Invitrogen) with 100 µL of LTX reagent and 50 µL of PLUS reagent per transfection.

Cells were collected 24 hpt in 1 mL of Pierce IP Lysis Buffer (Thermo Scientific) supplemented with protease inhibitor cocktail (MedChemExpress, HY-K0010) and 80 U/mL RNase inhibitor (Invitrogen RNaseOUT Recombinant Ribonuclease Inhibitor), and lysed by rotation at 4°C for 20 min. RIG-I-Flag was immunoprecipitated using Pierce Anti-DYKDDDK Magnetic Agarose (Invitrogen) according to the manufacturer’s instructions and eluted from the magnetic beads with Pierce 3× DYKDDDK Peptide (Thermo Scientific) supplemented with 80 U/mL RNase inhibitor (Invitrogen RNaseOUT Recombinant Ribonuclease Inhibitor). RNA co-purified with RIG-I-Flag was isolated using TRIzol reagent (Invitrogen).

Samples for immunoblot analysis were collected after the initial lysis step and after elution, then denatured by boiling in Laemmli sample buffer for 10 min. Proteins were separated by SDS-PAGE in 10% gels and visualized by Ponceau S staining to assess the purity of the pull-down samples. Proteins were transferred to nitrocellulose membranes, RIG-I-Flag was detected using mouse monoclonal anti-Flag M2 antibody (Sigma-Aldrich, F1804; 1:1,000 dilution), and SFV nsP1 was detected using in-house-generated rabbit polyclonal antiserum. LI-COR IRDye secondary antibodies were used to generate fluorescence signals, which were detected using the LI-COR Odyssey Fc Imaging System.

For Oxford Nanopore sequencing, samples were subjected to poly(A)-tail addition prior to library preparation with the cDNA-PCR Barcoding Kit V14 (SQK-PCB114.24, Oxford Nanopore Technologies). Sequencing was performed using the PromethION FLO-PRO114M flow cell (Oxford Nanopore Technologies). Sample A-tailing, library preparation, and sequencing were conducted by UAB SeqVision (Vilnius, Lithuania).

For short-read sequencing, libraries were prepared using the Illumina Stranded Total RNA Prep with Ribo-Zero Plus kit, omitting the rRNA depletion step, and sequenced on the NextSeq2000 P2 chip (Illumina) with 50 bp paired-end reads at the Institute of Genomics, University of Tartu.

### Statistical analysis

All data visualization and statistical analysis were performed with the GraphPad PRISM software. The *trans*-replicase assay data were analyzed for statistical significance using Student’s unpaired *t*-test and the IFN-β ELISA assay data with one-way ANOVA with a Dunnett’s post hoc test. *P* values below 0.05 were considered to represent statistically significant differences.

## Data Availability

All data used to reach the conclusions in the submitted paper and any data required to replicate the study findings are presented in the article and supplemental material (File S1 and S2). The sequences of all plasmids first reported in this study are provided in File S3. NGS data have been deposited in the NCBI SRA database under BioProject PRJNA1218509. All plasmids used in the study are available from authors without restrictions.

## SUPPLEMENTAL MATERIAL

File S1 jvi.02162-25-s0001.xlsx Raw data: measurements of Fluc and Gluc activities from *trans*-replicase assays used to generate images in Fig. 1, 3, 4, and 7.

File S2 jvi.02162-25-s0002.xlsx Raw data: measurements of rPAMP induced IFN-β levels by ELISA used to generate images in Fig. 2, 3, 4, 6, and 7.

File S3 jvi.02162-25-s0003.xlsx Sequence of plasmids CMV-P1234-WEEV, CMV-P1234^GAA^-WEEV, HSPolI-FG-WEEV, Ubi-P1234-WEEV, Ubi-P1234^GAA^-WEEV, AlbPolI-FG-WEEV, Ubi-P1234-EEEV, Ubi-P1234^GAA^-EEEV, AlbPolI-FG-EEEV, and pcDNA4/TO-RIG-I-Flag.

Supplemental legends jvi.02162-25-s0004.docx Descriptive legends for Files S1 to S3.

ASM does not own the copyrights to Supplemental Material that may be linked to, or accessed through, an article. The authors have granted ASM a non-exclusive, world-wide license to publish the Supplemental Material files. Please contact the corresponding author directly for reuse.
