# Supplementary material for: The ability of alphavirus replicases to synthesize non-viral type I interferon-inducing RNAs correlates with viral RNA synthesis and has a diverse impact on virus replication and pathogenicity
Source: J Virol. 2026 Jan 28;100(2):e02162-25. doi: 10.1128/jvi.02162-25 (PMC12911905; doi:10.1128/jvi.02162-25)
Supplement: Supplemental legends — Descriptive legends for Files S1 to S3. [file jvi.02162-25-s0004.docx]

**Supplemental legends.**

**Supplement 1.** Raw Data. Measurements of Fluc and Gluc activities from *trans*-replicase assays used to generate images on figures 1, 3, 4 and 7.

**Supplement 2.** Raw Data. Measurements of rPAMP induced IFN-β levels by ELISA used to generate images on figures 2, 3, 4, 6 and 7.

**Supplement 3.** Sequence of plasmids CMV-P1234-WEEV, CMV-P1234^GAA^-WEEV, HSPolI-FG-WEEV, Ubi-P1234-WEEV, Ubi-P1234^GAA^-WEEV, AlbPolI-FG-WEEV, Ubi-P1234-EEEV, Ubi-P1234^GAA^-EEEV, AlbPolI-FG-EEEV and pcDNA4/TO-RIG-I-Flag.
